# ATP binding to lysozyme and superfolder GFP amyloid fibrils induces aggregate remodeling and attenuates their cytotoxicity

**DOI:** 10.1038/s41420-026-03186-9

**Published:** 2026-06-04

**Authors:** Olga V. Stepanenko, Maksim I. Sulatsky, Kristina G. Gridasova, Ekaterina V. Mikhailova, Anna I. Sulatskaya, Olesya V. Stepanenko

**Affiliations:** 1 Laboratory of Structural Dynamics, Stability and Folding of Proteins, Institute of Cytology Russian Academy of Sciences, St. Petersburg, Russia; 2 Laboratory of Cell Morphology, Institute of Cytology Russian Academy of Sciences, St. Petersburg, Russia

**Keywords:** Neurodegeneration, Protein aggregation, Protein aggregation

## Abstract

The pathogenesis of neurodegenerative diseases such as Alzheimer’s and Parkinson’s, systemic and local amyloidoses is closely associated with amyloid fibril accumulation. Dysregulation of energy metabolism and a decline in the level of adenosine triphosphate (ATP), a key intracellular energy source and extracellular signaling molecule, contribute to the development of these pathologies. Despite the well-established role of ATP as a biological hydrotrope that prevents the initial aggregation of proteins, the impact of this nucleotide on the structure and properties of pre-formed (mature) amyloids is still poorly understood. In this study, we showed the ability of ATP to bind with affinity in the low-to-mid micromolar range to mature amyloids of lysozyme, which associated with hereditary lysozyme amyloidosis, and superfolder GFP, as model objects with unique properties, demonstrating a significant increase in the number of binding sites compared to native proteins. We demonstrated that this interaction induces declustering of both aggregate types, accompanied by fibril disordering specific to certain amyloidogenic proteins. Importantly, ATP-mediated fibril remodeling resulted in a significant reduction in their toxicity to mammalian cell lines, as well as a decrease in the aggregate resistance to chemical and thermal degradation. The obtained results reveal the complex nature of ATP’s effects: while acting as an endogenous amyloid detoxification factor, it is also susceptible to sequestration in amyloid deposits due to its affinity in the low-to-mid micromolar range. Amyloid-associated ATP sequestration may lead to nucleotide deficiency and aggravation of amyloidosis.

**Figure:**
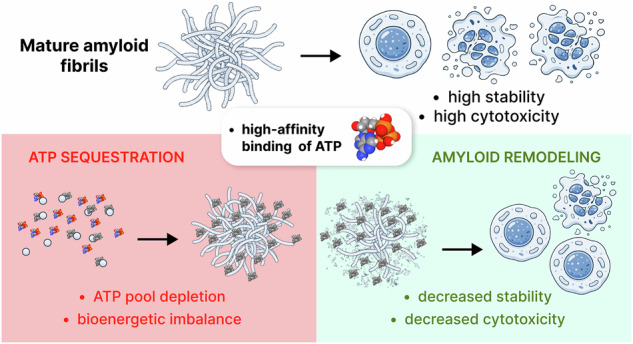

## Introduction

The pathogenesis of neurodegenerative diseases, such as Alzheimer’s [1], Parkinson’s [2] and amyotrophic lateral sclerosis [3], as well as systemic amyloidoses [4], is inextricably linked to the accumulation of amyloid fibrils. The amyloid fibril formation takes place against the backdrop of cellular aging and mitochondrial impairment, which result in a dramatic decrease in the level of adenosine triphosphate (ATP) [5]. It is known that ATP depletion disrupts the function of protein quality control systems, such as chaperones and proteasomes, leading to the accumulation of aberrant aggregates [6, 7]. Moreover, ATP deficiency affects the physical attributes of intracellular non-membranous organelles implicated in RNA metabolism and the stress response, likely owing to the suppression of ATP-driven helicases within their structure [8]. The disruption of the process of biogenesis of non-membranous organelles can result in the aggregation of proteins that are involved in their formation, including the generation of amyloid fibrils [9].

Recent studies have revealed a new role for ATP as a biological hydrotrope: at millimolar concentrations (intracellular level is in the range 1–10 mМ, depending on the type of tissue and cell [10–13]), this nucleotide is capable of directly interfering with the process of phase separation and initial aggregation of proteins, independent of its function as an energy source [14, 15]. The influence of ATP on the initiation and progression of fibrillogenesis of various amyloidogenic peptides has been extensively studied, leading to the accumulation of extensive knowledge in this field [16–21]. At the same time, the interaction between the nucleotide and pre-existing (mature) amyloid fibrils remains a subject of limited understanding. This aspect is crucial for understanding the spatiotemporal dynamics of amyloidosis in vivo. For example, extracellular amyloid deposits may initially form and mature within the extracellular matrix, where basal levels of extracellular ATP are in the sub-micromolar range [22]. However, as cytotoxic amyloid plaques grow, damage to surrounding tissues and cell lysis lead to the release of intracellular ATP and the formation of local microdomains with a high concentration of the nucleotide acting as a danger signal (DAMP) [23, 24]. Similarly, intracellular aggregation of proteins such as alpha-synuclein [25], TDP-43 [26], and tau [27] often occurs under conditions of impaired energy metabolism and reduced levels of ATP, which is characteristic of aging and a number of neurodegenerative diseases [28, 29]. In these scenarios, amyloid structures may form under conditions of nucleotide deficiency, which also raises the question of their subsequent interaction with metabolites during the later stages of the pathological process. Thus, mature amyloid aggregates are long-lived supramolecular structures that can persist for a long time in tissues and interact with various components of the cellular environment [30, 31]. Despite this, the exact role of cellular metabolites such as ATP on the structure and properties of pre-formed amyloid fibrils remains poorly understood. This is a critical gap, as clinical practice often encounters with pre-formed deposits. The ability of ATP to modulate the cytotoxicity of mature aggregates, as well as the underlying mechanisms, remains an area that is not yet well understood.

In this study, we undertake for the first time a comprehensive analysis of the interplay between ATP and mature fibrils formed from globular proteins. In order to assess the universality of the observed effects and identify the dependence on the type of amyloid, two model systems were employed, each with distinct structural properties: pathological lysozyme fibrils accumulating in systemic lysozyme amyloidosis [32, 33] and model amyloids based on the superfolder GFP (sfGFP), the green chromophore of which serves as an internal marker for spectroscopic detection of structural changes in the fibrillar backbone [34, 35]. Due to the high stability and reproducibility of their structure and properties, amyloid fibrils of lysozyme and sfGFP represent convenient model objects for biophysical research, including studies of fibril remodeling. Employing these model systems, we conducted an analysis of the interaction between ATP and the fibrillar backbone, examining the resulting structural alterations, and delineated a correlation between morphological modifications and the cytotoxic effects of amyloids on mammalian cells, as well as the resistance of these aggregates against chemical and thermal denaturation. We used a fairly wide range of micromolar concentrations of ATP. This allowed us to ensure the required level of both intracellular and extracellular nucleotide under physiological and pathological conditions. Our data suggest that, in addition to its well-established role in preventing the aggregation of intracellular proteins, ATP also has the capacity to counteract the cytotoxicity associated with mature amyloid deposits.

## Results

### Binding affinities of ATP to amyloid fibrils

**Fig. 1:**
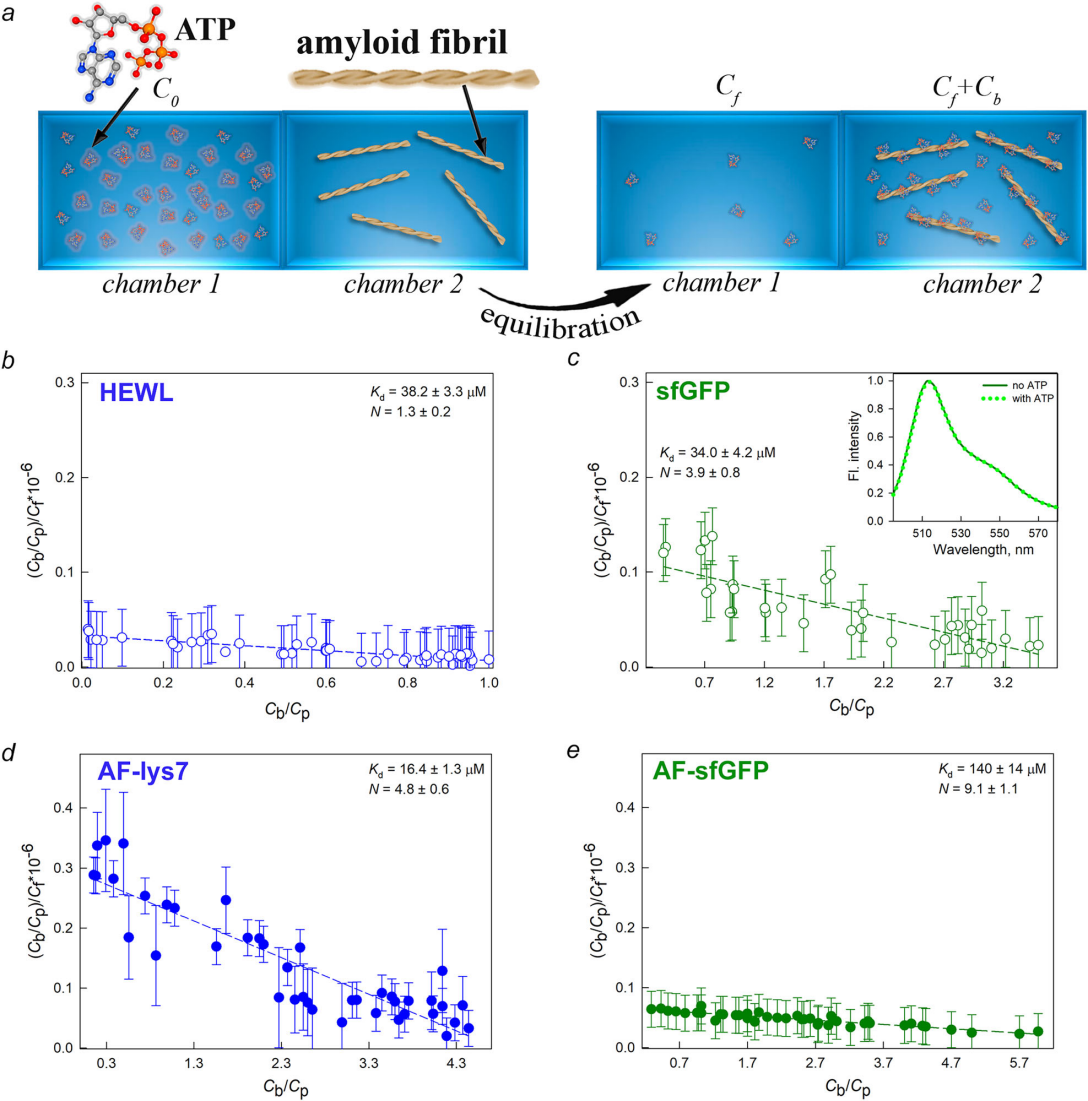
Binding of ATP to proteins in their native and amyloid forms. **a** Schematic representation of an equilibrium microdialysis device. It consists of two chambers of equal volume, separated by a membrane that is impermeable to large particles. А sample of amyloid fibrils of known concentration and ATP (initial concentration *C*_0_) is placed in different chambers of the device (left image). After equilibration, provided that ATP binds to the sample, the amount of ATP in the sample chamber will exceed the amount of free nucleotide (*C*_f_) by its amount in the bound state (*C*_b_). After spectroscopic analysis of solutions obtained by equilibrium microdialysis, the data can be presented as Scatchard plots. Scatchard plot for the interaction of ATP with native proteins, hen egg white lysozyme (HEWL; **b**) and super folder GFP (sfGFP; **c**), and amyloid fibrils formed from lysozyme (AF-lys7; **d**) and sfGFP (AF-sfGFP; **e**). The experimental data (circles) and best fit curve with the binding constants (*K*_d_) and the number of binding sites (*N*) are shown. (insert to **c**) The effect of ATP on the green chromophore fluorescence intensity of sfGFP in native form. *λ*_ex_ = 490 nm.

To test the ability of ATP to interact with mature amyloids, we decided to use absorption spectroscopy of samples prepared by equilibrium microdialysis [36] (Fig. 1a). To verify the correctness of the results obtained using the proposed technique, we first investigated the interaction of ATP with lysozyme and sfGFP in their native forms (Fig. 1b, c). The linearity of the Scatchard plots constructed for lysozyme and sfGFP in their native forms upon ATP binding was noted (Fig. 1b, c). This suggests that all ATP binding sites on globular proteins are identical and have the same binding characteristics. In this regard, the Scatchard plot was analyzed under the assumption of the existence of one binding mode. It turned out that native lysozyme has a single, highly specific ATP binding site with binding constant in the micromolar range, while native sfGFP has four ATP binding sites per protein molecule with affinity in the low-to-mid micromolar range (Fig. 1b, c). It is worth noting that the detection of a single ATP binding site at micromolar concentrations on the surface of native lysozyme is in line with the published data on the interaction between this protein and nucleotides [14]. This implies that the proposed approach can be used to determine the number of binding modes and parameters of ATP binding to proteins in different states, especially in the fibrillar state. It should be noted that we did not detect any changes in the fluorescent signal of sfGFP in the UV or visible region (Fig. 1c, inset) when it was turned on with ATP, indicating that the nucleotides bind to the beta-barrel protein away from the chromophores, tryptophan, and green chromophore protein. It is worth noting that we failed to detect any alterations in the fluorescent signal of sfGFP in the UV or the visible spectral region during its interaction with ATP (Fig. 1c, inset), which suggests that the nucleotides bind to the beta-barrel of the protein at a distance from the chromophores, tryptophan, and the green chromophore.

Following the successful validation of the method, it was employed to demonstrate the feasibility of ATP interaction with mature amyloids (Fig. 1d, e). A wide range of micromolar concentrations of ATP were used in the study. The results, presented in the form of Scatchard plots, indicate that ATP binds to the amyloids in both samples, and the linearity of these dependencies suggests the existence of a single mode of ATP binding to fibrils (Fig. 1d, e). The binding parameters of ATP to amyloid fibrils based on lysozyme and sfGFP were determined. It was demonstrated that in the case of both amyloids, the dissociation constant for ATP is within the micromolar concentration range, indicating a relatively strong binding affinity. However, the key difference between the interaction of ATP with the monomers and fibrils of the studied proteins was a multiple increase in the number of binding sites per monomer: it increased to 5 for lysozyme amyloids and to 9 for sfGFP amyloids.

Thus, for the first time, we have demonstrated the low-to-mid micromolar affinity of ATP interaction not only with native proteins but also with amyloid fibrils derived from them, and we have quantified this interaction. It has been demonstrated that the transformation of a protein from its native to aggregated state is not associated with any loss, but rather results in the appearance of novel nucleotide-binding sites, suggesting the specific integration of ATP into the architecture of the amyloid fibril.

### The effect of ATP binding on the morphology and size of amyloid fibril clusters

Literature suggests that ATP can alter the properties of amyloid structures [19] formed in its presence and stabilize the native protein structure [14]. In this context, we hypothesized that the association of ATP with mature amyloid fibrils may influence their characteristics. To explore the impact of ATP binding on the properties of mature amyloids, we examined the alterations in the size, morphology, structure, as well as stability and cytotoxicity of lysozyme and sfGFP amyloids following the addition of ATP

**Fig. 2:**
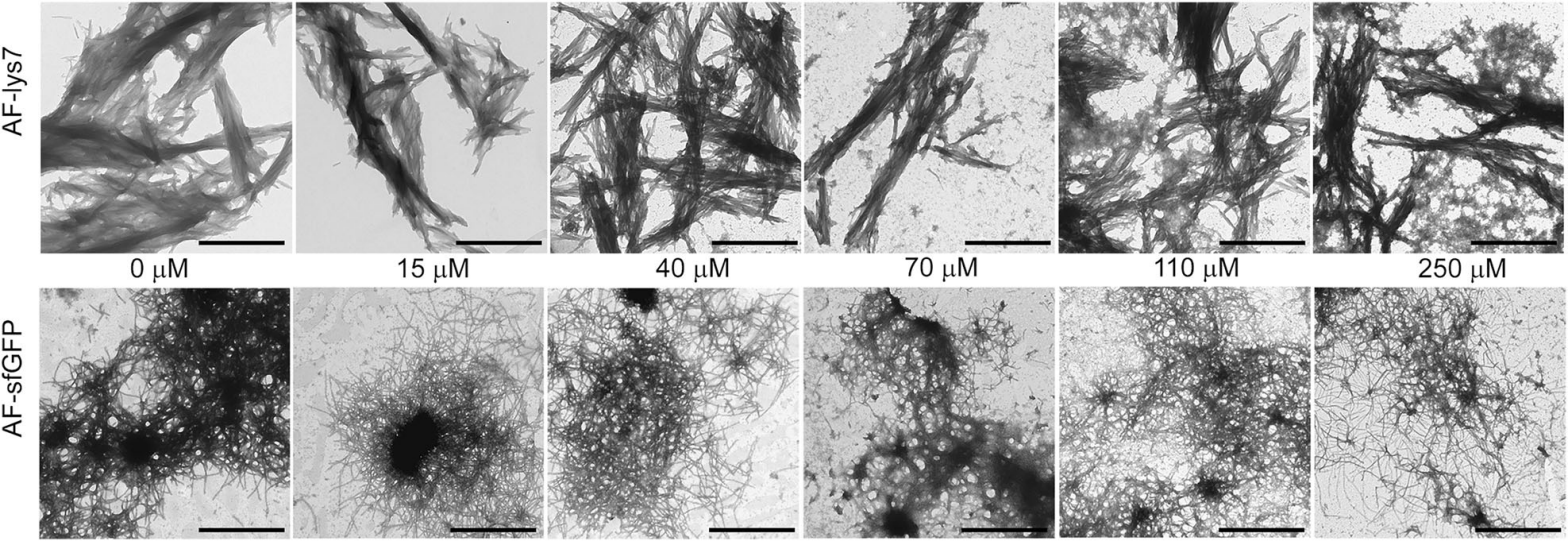
ATP-induced changes in amyloid fibril morphology. Visualization of amyloids using transmission electron microscopy (TEM) in the ATP absence (0 µM) and with increasing nucleotide concentration (numbers next to the micrographs represent ATP concentration). Microphotographs of AF-lys7 and AF-sfGFP are shown in the top and bottom rows, respectively. Scale bars are equal to 1 µm.

First, we analyzed whether the binding of ATP to amyloids causes a change in their morphology using the transmission electron microscopy method (TEM) (Fig. 2). The fibrillar structure of control samples of amyloids based on lysozyme and sfGFP (i.e., in the absence of ATP) was monitored at the beginning and end of the experiment, which allowed us to conclude that the structure remained unchanged (Fig. S1a). This allowed us to establish that the alterations we detected in amyloids were attributable to ATP binding.

In the control sample, lysozyme amyloids were tightly packed and organized into large bundles (Fig. 2). The incorporation of ATP in varying amounts into the fibril suspension resulted in two outcomes. First, with a moderate increase in ATP concentration (up to 40 μM, Fig. 2, top row), a marked decrease in the density of fibril clusters was observed, which led to a clear visualization of the boundaries of individual fibers. Secondly, a further increase in ATP concentration (>70 μM, Fig. 2) resulted in the loosening and loss of the fibrillar morphology of lysozyme amyloid: first in the peripheral regions of the clusters, and then over more extensive areas. Thus, we observed declustering of lysozyme amyloid fibrils and disruption of their structure when they interacted with ATP at micromolar concentrations.

Intact sfGFP amyloids were also present as large, dense clusters in the form of tangles, surrounded by individual fibrils. This is different from the clustering pattern of lysozyme fibrils (see Fig. 2, bottom row). In the presence of ATP, we observed a reduction in the size and density of fibrillar clots of sfGFP. Notably, the declustering effect of ATP on sfGFP amyloids was more pronounced than on lysozyme amyloids: at maximum ATP concentrations (250 μM, Fig. 2), we visualized a large number of individual fibers.

**Fig. 3:**
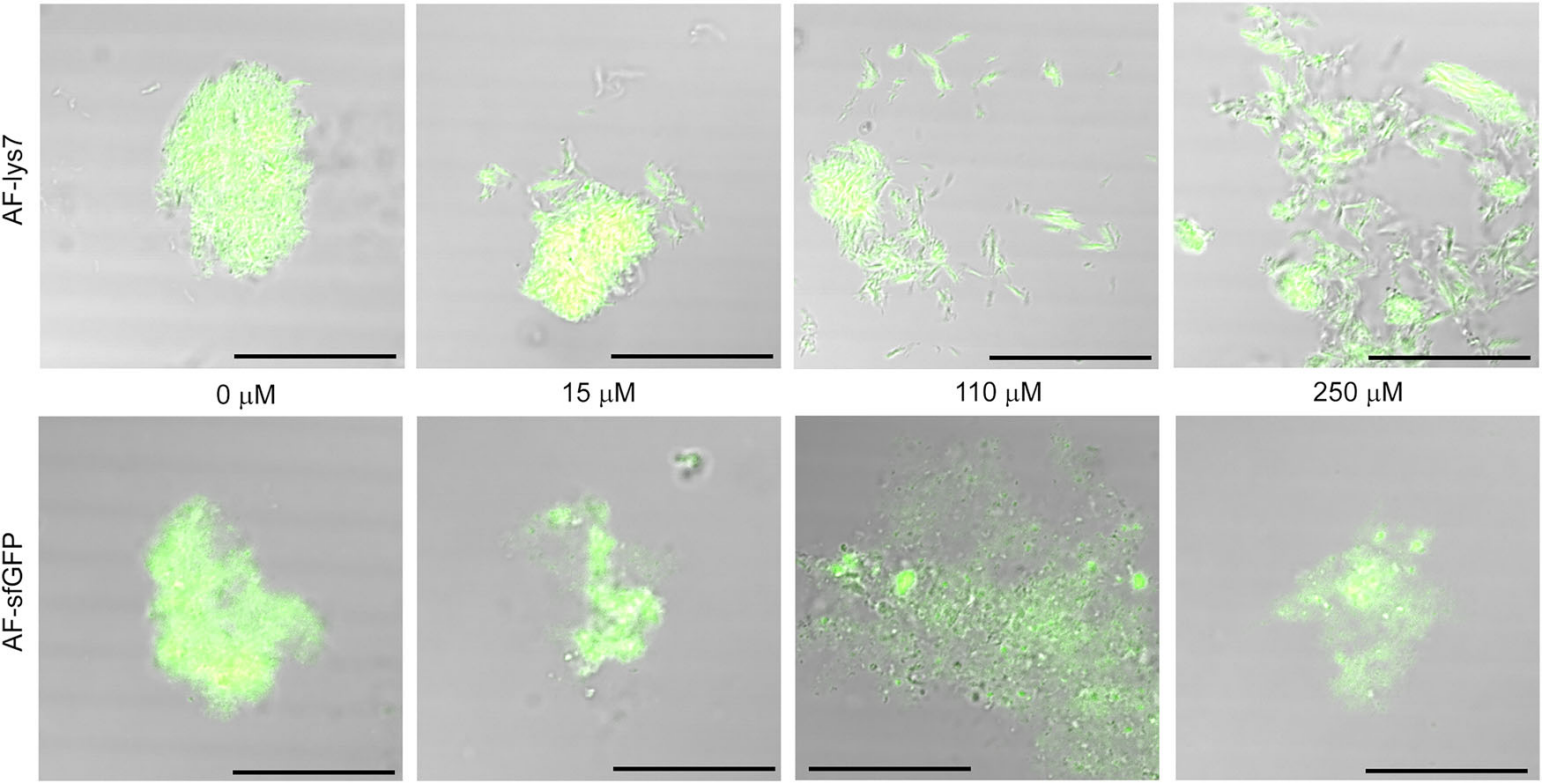
ATP-induced changes in amyloid fibril clustering. Visualization of amyloids using confocal laser scanning microscopy (CLSM) in the ATP absence (0 µM) and with increasing nucleotide concentration (numbers next to the micrographs represent ATP concentration). An overlay of fluorescence of ThT added to AF-lys7 (the top row) and AF-sfGFP (the bottom row) and transmitted light images are present. Scale bars are equal to 20 μm.

**Fig. 4:**
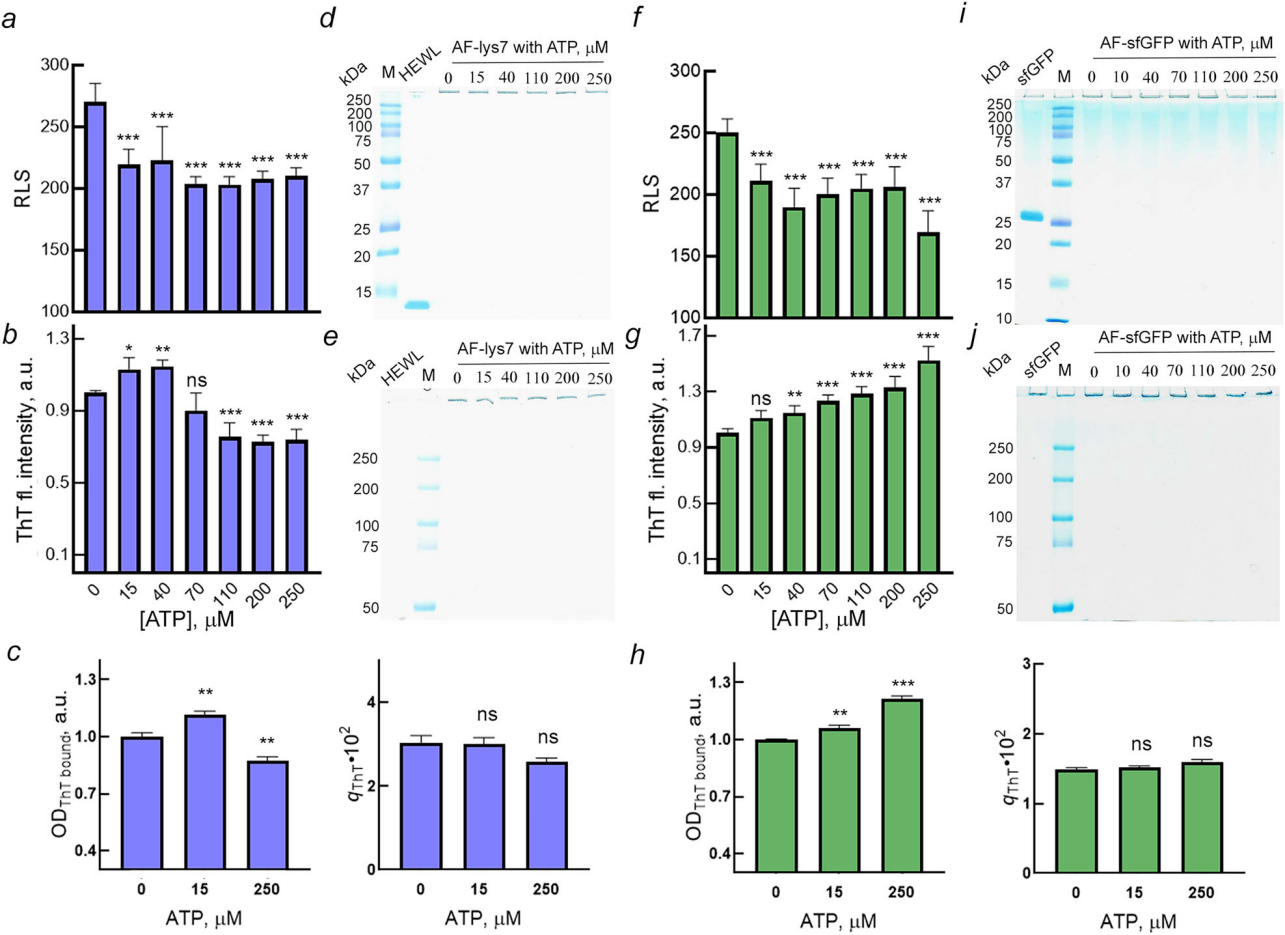
ATP-induced changes in the size of amyloid fibrils. The value of Rayleigh Light Scattering (RLS) of AF-lys7 (**a**) and AF-sfGFP (**f**). Integral fluorescence intensity of ThT added to samples of AF-lys7 (**b**) and AF-sfGFP (**g**), *λ*_ex_ = 440 nm. The relative amount (monitored by absorbance at 440 nm; data are normalized to that for amyloid without any treatment) and fluorescence quantum yield of amyloid-bound ThT determined for the samples prepared by equilibrium microdialysis. Samples of AF-lys7 (**c**) and AF-sfGFP (**h**) were tested in the absence of any treatment, as well as treated with 15 or 250 μM ATP. Pseudo-native 17% (**d**, **i**) and 8% (**e**, **j**) SDS-PAGE of AF-lys7 (**d**, **e**) and AF-sfGFP (**i–j**). Amyloid fibrils in the ATP absence (0) and treated with increasing nucleotide concentration (ATP concentration in µM is indicated above the gel images) were tested. Corresponding monomeric protein (HEWL or sfGFP) at a concentration equal to that of intact amyloids was loaded as a control. Marker proteins (M) of known molecular weights (numbers on the left) were loaded. Data in bar graphs are presented as mean ± SD, based on five replicates. ***p < 0.001, **p < 0.01, *p < 0.05; and ns *p* ≥ 0.05.

The declustering effect of ATP binding on the analyzed amyloids was also confirmed by confocal laser scanning microscopy (CLSM) of samples in the presence of ThT (Fig. 3). Indeed, large, dense, and uniformly stained ThT amyloid clusters in the absence of nucleotides, when ATP was added, became less dense and disaggregated in the case of lysozyme amyloid, and looser and less intensely stained in the case of sfGFP amyloids. The observed changes in the size of lysozyme and sGFP amyloids clusters as a result of ATP binding were also confirmed by a decrease in Rayleigh light scattering (RLS) of the samples (Fig. 4a, f). It is important to note that, despite the apparent destruction of the clusters, the analysis by pseudo-native SDS-PAGE on 17% and 8% gel (Fig. 4d, e, i–j) did not reveal the presence of monomeric or oligomeric protein forms (up to 250 kDa). This observation suggests that ATP acts as a declustering factor (separating fibrils), yet it does not result in the complete disassembly of the amyloid core into individual monomers.

Before further analysis of the structure of amyloid fibrils in the presence of ATP, it was necessary to ensure that the effects we observed were associated with the specific binding of the nucleotide by amyloids and not caused by changes in environmental conditions. In particular, the addition of ATP and its spontaneous hydrolysis can lead to a change in the ionic strength of the solution. Control experiments showed no structural or morphological changes in the analyzed amyloid fibril samples (Fig. S2) in the presence of 1 mM NaCl (which corresponds to the expected change in ionic strength based on the ionic equilibrium in the ATP-Mg solution at a neutral pH [37] and the expected rate of nucleotide hydrolysis [38]).

To further analyze the effect of ATP on the structure of both amyloids, we recorded the integrated fluorescence intensity of the amyloid-specific dye ThT added to the studied samples. This characteristic depends both on the length/number of fibrils (i.e., on the amount of dye bound to the fibrils) and on the structure of the amyloid fiber (i.e., on the quantum yield of the dye), which makes it possible to study the degradation of amyloid fibrils using it [39]. To test the correctness of this approach, a control experiment was first conducted, in which we verified that ThT and ATP do not compete for binding sites with fibrils. To do this, we added ThT and ATP to the amyloid suspension in different orders and then compared the ThT fluorescence intensity in the samples. It turned out that the intensity of ThT fluorescence in the lysozyme amyloid suspension did not depend on the order of dye addition to the sample (Fig. S1, b), which suggests that ATP and ThT interact with different sites on the surface of fibrils without competing for binding sites.

In the case of sfGFP amyloids, the ThT fluorescence intensity increased linearly with increasing ATP concentration (Fig. 4g). It should be noted that the fluorescence intensity value of ThT in the presence of ATP alone (even at maximal applied concentration) coincides with that of free ThT in the buffered solution, and both values are significantly lower than the fluorescence intensity value of ThT in the presence of amyloid samples with or without ATP (Fig. S3). This means that ATP in the concentration range used does not affect the spectral characteristics of ThT. Linear increase in the fluorescence intensity of the dye in the presence of ATP-treated sfGFP amyloids can be explained by an increase in the surface of fibrils available for dye binding due to their release from the depths of dense clots. As suggested by the TEM results for sfGFP amyloids and further confirmed by the characterization of the amyloid structure (see next paragraph), the fiber structure remained unchanged in the presence of ATP. Due to the disintegration of sfGFP amyloid clusters, more dye molecules bind to amyloids, leading to an increase in the ThT fluorescence intensity. This is also confirmed by the increase in the amount of bound ThT determined for ATP-treated amyloids relative to the amount of dye bound to intact amyloids without changing the quantum yield of the bound dye (Fig. 4h). In contrast, a two-stage change was observed for lysozyme amyloids: an increase in the signal up to 40 μM ATP was followed by a decrease (Fig. 4b). It is likely that the initial disruption of clusters increases the accessibility of sites for ThT binding, but subsequent structural disorganization either decreases the ability of fibrils to bind the dye (due to disruption of fibrillar core) or affects the quantum yield of ThT (due to reorganization of fibrillar core). According to TEM, “loosening” of the backbone of lysozyme amyloids can be considered as preferential scenario, which is also confirmed by the structure analysis (see next paragraph). Indeed, the amount of ThT bound to amyloids increased when amyloids were exposed to low concentrations of ATP, and decreased when the ATP concentration increased, while no significant changes in the quantum yield of the bound dye were observed (Fig. 4c).

Thus, ATP exerts a declustering effect universally across different amyloid types, but the possibility of subsequent structural reorganization depends on the nature of the precursor protein.

### The effect of ATP binding on the structure of amyloid fibrils

**Fig. 5:**
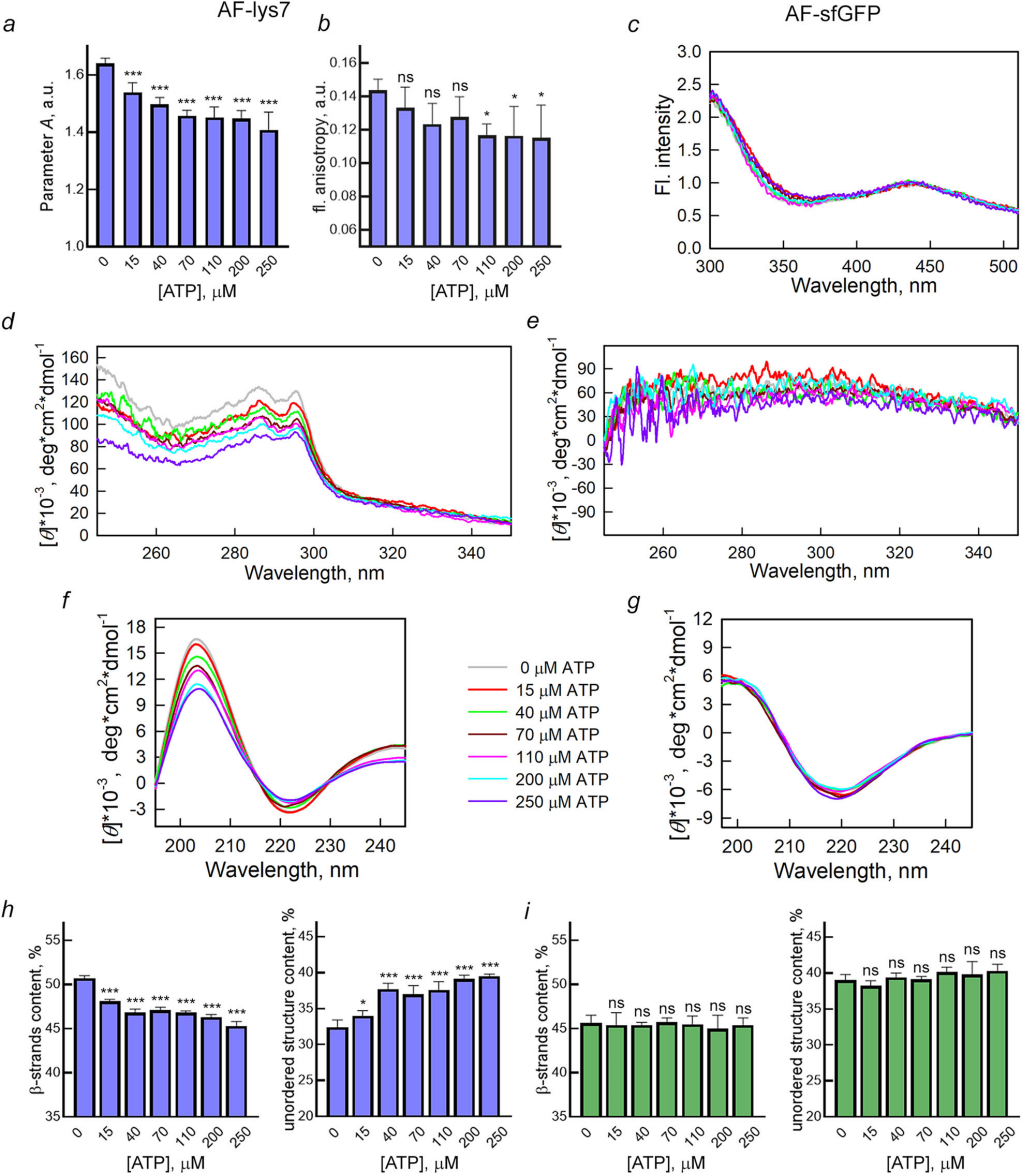
ATP-induced structural changes in amyloid fibrils. Parameter *A* (ratio of fluorescence intensity at 320 and 365 nm wavelengths, the value of which is sensitive to the microenvironment of tryptophan residues), *λ*_ex_ = 295 nm (**a**) and fluorescence anisotropy (*λ*_ex_ = 295 nm, *λ*_em_ = 365 nm; **b** of AF-lys7. **c** Fluorescence spectra of aromatic residues and the specific chromophore of AF-sfGFP, *λ*_ex_ = 280 nm. Near-UV CD spectra of AF-lys7 (**d**) and AF-sfGFP (**e**). Far-UV CD spectra of AF-lys7 (**f**) and AF-sfGFP (**g**). Secondary structure of AF-lys7 (**h**) and AF-sfGFP (**i**) estimated by deconvolution of far-UV CD spectra by BeStSel algorithm [102]: β-sheets and unordered structure are shown. Data in bar graphs are presented as mean ± SD, based on five replicates. ***p < 0.001, **p < 0.01, *p < 0.05; and ns *p* ≥ 0.05.

To understand the molecular mechanisms behind the changes in the structure of both types of amyloids upon binding to ATP, we also characterized the samples using fluorescence spectroscopy and circular dichroism techniques (Fig. 5). In the case of lysozyme amyloid fibrils, as a protein containing tryptophan, the fluorescence anisotropy and the parameter *A* (the ratio of fluorescence intensity at 320 and 365 nm) were measured in the samples to analyze changes in the polarity and packing density of aromatic protein residues within the fibrils. In the case of amyloids based on sfGFP bearing a “green” chromophore, we analyzed a change in the shape of a specific fluorescence band in the spectra of this amyloids [34].

The values of parameter *A* and the anisotropy of fluorescence of lysozyme amyloid fibrils decreased as the ATP concentration increased (Fig. 5a, b). Furthermore, there was a reduction in the intensity of the near-UV circular dichroism signal for lysozyme fibrils when exposed to ATP, suggesting a diminished chiral environment for aromatic amino acids within these amyloid structures (Fig. 5d). Taken together, these findings suggest that the interaction between amyloids and nucleotides leads to the loosening of the hydrophobic core in fibrils and the reorganization of regions containing aromatic residues.

The change in the far-UV CD spectra of lysozyme fibrils upon ATP binding (Fig. 5f) indicated a partial transformation of their secondary structure. The analysis of these spectra using the BestSel method revealed that these alterations correlate with a reduction in the proportion of β-strands in lysozyme amyloid structures, which is accompanied by an increase in the content of disordered structure (see Fig. 5h). This confirms the ability of ATP to induce partial melting of the rigid β-structural backbone of lysozyme fibrils.

A radically distinct picture was observed in the case of sfGFP amyloids. The addition of ATP did not lead to a significant change in the fluorescence spectra at 280 nm excitation (Fig. 5c) or the near-UV (Fig. 5e) and the far-UV (Fig. 5g) CD spectra. The analysis of the secondary structure content reveals the invariance in the proportion of β-strands and disordered structures of the sfGFP amyloids when it interacts with ATP (Рис. 5, *i*).

These data allow us to draw an important conclusion: declustering of sfGFP amyloids as a result of their interaction with ATP, previously revealed by visualization methods (TEM and CLSM), proceeds without destroying the secondary structure of the individual fibrils. At the same time, for lysozyme amyloids, the action of ATP is more profound and affects the very structure of the protein within the fiber. This is consistent with the data on the RLS and ThT fluorescence changes, confirming that the mechanism of ATP action can vary from preferential declustering (as for sfGFP) to deep structural reorganization of the fibrillar backbone (as for lysozyme) depending on the type of precursor protein.

#### The effect of ATP binding on the resistance of amyloid fibrils to physico-chemical influences

**Fig. 6:**
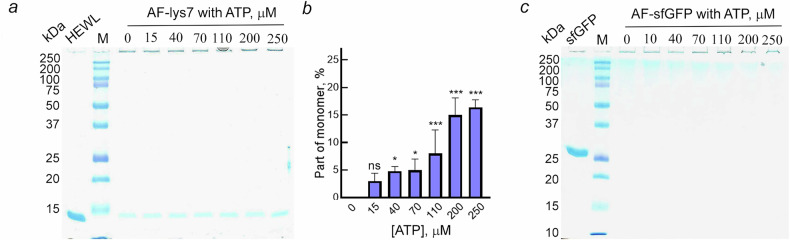
ATP-induced changes in stability of amyloid fibrils. Evaluation of resistance to ionic detergents and boiling of AF-lys7 (**a**) and AF-sfGFP (**c**) by 17% SDS-PAGE. Amyloid fibrils in the ATP absence (0) and treated with increasing nucleotide concentration (ATP concentration in µM is indicated above the gel images) were tested. Corresponding monomeric protein (HEWL or sfGFP) at a concentration equal to that of intact amyloids was loaded as a control. Marker proteins (M) of known molecular weights (numbers on the left) were loaded. **b** The quantitative content of protein monomer in AF-lys7 samples after treatment with ionic detergents and boiling was assessed using ImageJ. The intensity of the lysozyme monomer bands in amyloid samples in the absence and presence of ATP was normalized to the intensity of the native monomeric protein band. Since amyloids in the nucleotide absence were partially reduced to monomers upon exposure to ionic detergent and temperature, the amount of monomeric protein in this sample was used as a baseline for assessing monomer increment after exposure to ATP. Data in bar graphs are presented as mean ± SD, based on five replicates. ***p < 0.001, **p < 0.01, *p < 0.05; and ns *p* ≥ 0.05.

The ability of ATP to loosen the fibril structure raised the question of the influence of the nucleotide on their resistance to denaturing action, which was analyzed by SDS-PAGE under denaturing conditions. It turned out that lysozyme amyloids, which demonstrated high resistance to boiling with 2% SDS in the control (only a faintly colored band corresponding to the protein monomer was visualized on the gel), became sensitive to physicochemical degradation effects in the presence of ATP: a dose-dependent increase in the fraction of monomers was observed on the gel (Fig. 6a, b). This indicates the destabilization of the intermolecular interactions that stabilize the fibrillar backbone of lysozyme.

In contrast, amyloids of sfGFP maintained high resistance to SDS and boiling, even in the presence of maximum concentrations of ATP (Fig. 6c). The lack of increase in the proportion of monomers for sfGFP may be due to the fact that in this case, ATP effectively breaks the bonds between fibrils, but does not disrupt the rigid packing of the beta structure within an individual fiber.

Thus, although ATP efficiently declusters both types of amyloid fibrils, a decrease in their resistance to chemical and thermal denaturation was observed only in lysozyme fibrils, while sfGFP fibrils retained high stability of their backbone.

### The impact of ATP binding on the cytotoxic effect of amyloid fibrils on immortalized cell lines

**Fig. 7:**
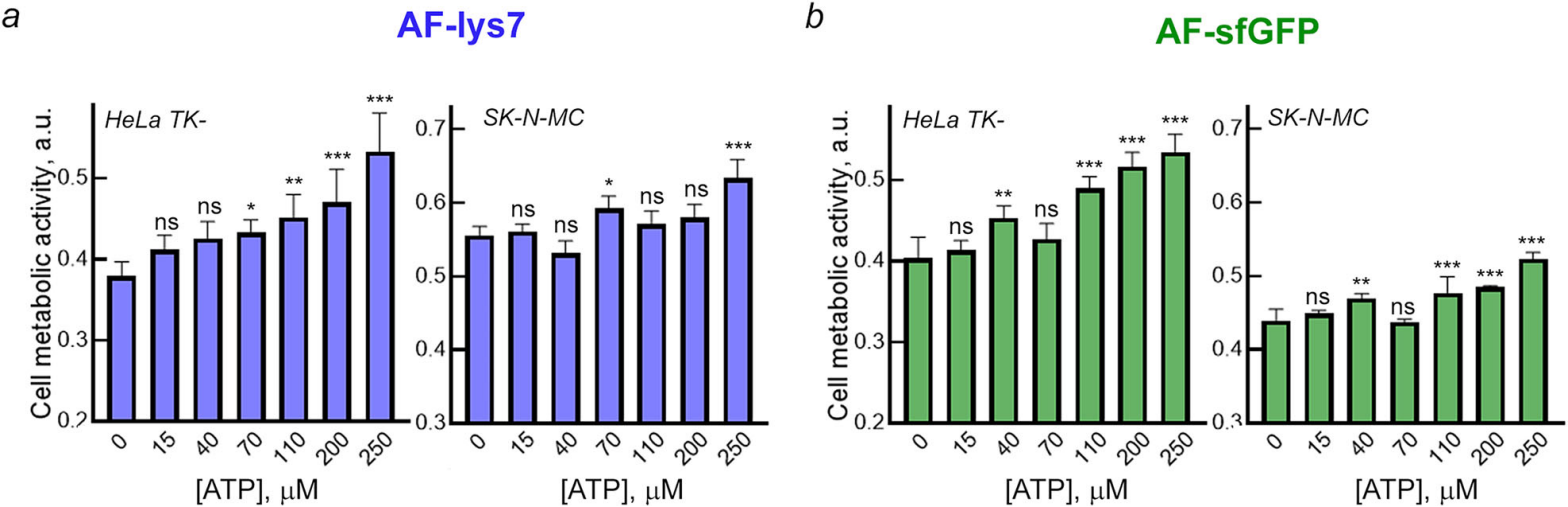
Changes in cytotoxicity of lysozyme and sfGFP amyloid fibrils as result of ATP action. Relative metabolic activities of two cell lines, cervical epithelioid carcinoma (HeLa TK-) and neuroblastoma (SK-N-MC), after 24 h exposure to AF-lys7 (**a**) and AF-sfGFP (**b**) are presented. All values are normalized to the metabolic activity of control cells treated with non-fibrillar protein in the same buffer and at the same concentration as the amyloid fibrils. Data are presented as mean ± SD, based on five replicates. ***p < 0.001, **p < 0.01, *p < 0.05; and ns *p* ≥ 0.05.

The final stage of the work involved assessing the biological significance of the effects that were detected. The toxicity of lysozyme and sfGFP amyloids, after their interaction with ATP, was evaluated against mammalian cells from two different lines: human cervical epithelioid carcinoma (HeLa TK-) and neuronal epithelioma (SK-N-MC) (Fig. 7).

In the absence of nucleotides, amyloids caused a significant decrease in the metabolic activity of cells, as shown by the MTT assay (Fig. 7). However, preincubating fibrils with ATP (up to 250 µM) resulted in a dose-dependent increase in cell survival (Fig. 7). It is crucial to highlight that ATP alone, at a concentration of up to 500 μM, did not impact the metabolic activity of cells (Fig. S4), allowing us to confidently attribute the observed protective effect to the ATP-mediated detoxification of amyloid structures.

Thus, disintegration of clusters and structural “softening” of fibrils under the influence of ATP directly correlate with the loss of their cytotoxic potential.

## Discussion

### ATP binds to amyloids with the low-to-mid micromolar affinity, leading to their declustering, disordering, and detoxification

In this study, we investigated the ability of ATP to specifically bind to various amyloid fibrils, using the examples of pathological lysozyme amyloids accumulated in systemic lysozyme amyloidosis and model sfGFP amyloids, and, for the first time, determined the binding parameters of ATP to the amyloids under study.

It should be noted that the number of ATP binding sites for lysozyme and sfGFP in their native form differs several times (one for lysozyme versus four for sfGFP), although the nucleotide binding constant for them is almost the same. Certainly, owing to the extended length of its polypeptide chain, sfGFP may statistically possess a greater number of potential nucleotide-binding sites. Additionally, we cannot ignore the distinctive architectural characteristics of proteins: unlike lysozyme, sfGFP possesses a unique spatial configuration, known as a beta barrel, the cavity of which can facilitate the formation of additional ATP binding sites. At the physicochemical level, the number of ATP binding sites is determined by the availability of aromatic and positively charged groups of the protein, which ensure coordination and the formation of specific bonds with the nucleotide. For globular lysozyme, the specificity of interaction with ATP is shown [14] to arise from stacking and π-cation contacts of the nucleotide triphosphate fragment with the protein aromatic pocket and Arg 61, being additionally stabilized by non-covalent interactions of the adenosine fragment of the nucleotide with Leu 75, Asp101, and Asn10. In this case, the nucleotide binding site partially overlaps with the active site of the enzyme. Apparently, globular compact highly stable monomeric lysozyme - an enzyme with highly specialized function of hydrolysis - has a more limited availability of potential nucleotide-binding sites compared to sfGFP, whose spatial organization in the form of a β-barrel is necessary for localization inside the green chromophore and control of its photophysical properties [40, 41]. Therefore, the disparity in the number of ATP binding sites between monomeric lysozyme (one site) and sfGFP (four sites) can be attributed to the size, structural differences, and functional objectives of the proteins, which collectively determine the distinct availability and specificity of sites for interaction with ATP.

We demonstrated that lysozyme and sfGFP in their fibrillar form retain an ATP binding constant of the micromolar order, but exhibit a multiple increase in the number of binding sites for nucleotides compared to their native forms. Importantly, micromolar affinities are common in biological regulation and are typical for dynamic interactions within signaling and metabolic networks, where reversibility and sensitivity to ligand concentration are required [42, 43]. Furthermore, interpretation of the measured *K*_d_ values should consider the supramolecular nature of amyloid fibrils. These structures represent extended polymeric aggregates with repeating structural motifs and numerous potential interaction sites exposed on their surface. In such multivalent systems, the effective binding strength is determined not only by the affinity of a single binding site but also by the overall avidity of the structure arising from the high density of interaction sites on the fibril surface [30, 44]. Notably, in our experiments, measurable changes in the structural and biophysical properties of the studied amyloid aggregates were already observed at low micromolar concentrations of ATP (Figs. 4 and 5) and increased progressively with increasing nucleotide concentration. These results indicate that even relatively low ATP levels can induce detectable structural remodeling of lysozyme and sfGFP amyloid fibrils. Taken together, these observations indicate that moderate affinity at the level of individual binding sites translates into effective ATP association with the amyloid structures tested due to the multivalent nature of the fibrillar surface.

An analysis of the morphology, structure, and stability of amyloid fibrils formed from lysozyme and sfGFP allows us to conclude that ATP binding has a significant declustering effect on them. This finding is consistent with existing literature data, which suggest that ATP can disintegrate the fibrils from the intrinsically disordered prion NM domain *Saccharomyces cerevisiae* Sup35 in vitro, resulting in the formation of individual protofibrillar structures without significant changes in their number or molecular weight [19]. It is interesting to note that, according to the results of this study, the formation of a more compact fibrillar structure increased the resistance of the aggregates to the effects of ATP. These data are in agreement with the findings of our study. Indeed, we also observed increased resistance to the declustering effect of ATP on lysozyme amyloids, which formed more densely packed clusters of amyloid fibers with a longer β-structural backbone compared to sfGFP amyloids.

In addition to the declustering effect observed for both amyloid types, we also found that ATP could reduce the ordering of amyloid fibers depending on the type of amyloid fibrils: the disordering effect of ATP was more pronounced for lysozyme amyloids, while sfGFP amyloids remained structurally stable. This allowed us to hypothesize that the ability of ATP to destroy the amyloid fibrils tested correlates with the affinity of its interaction with them.

**Fig. 8:**
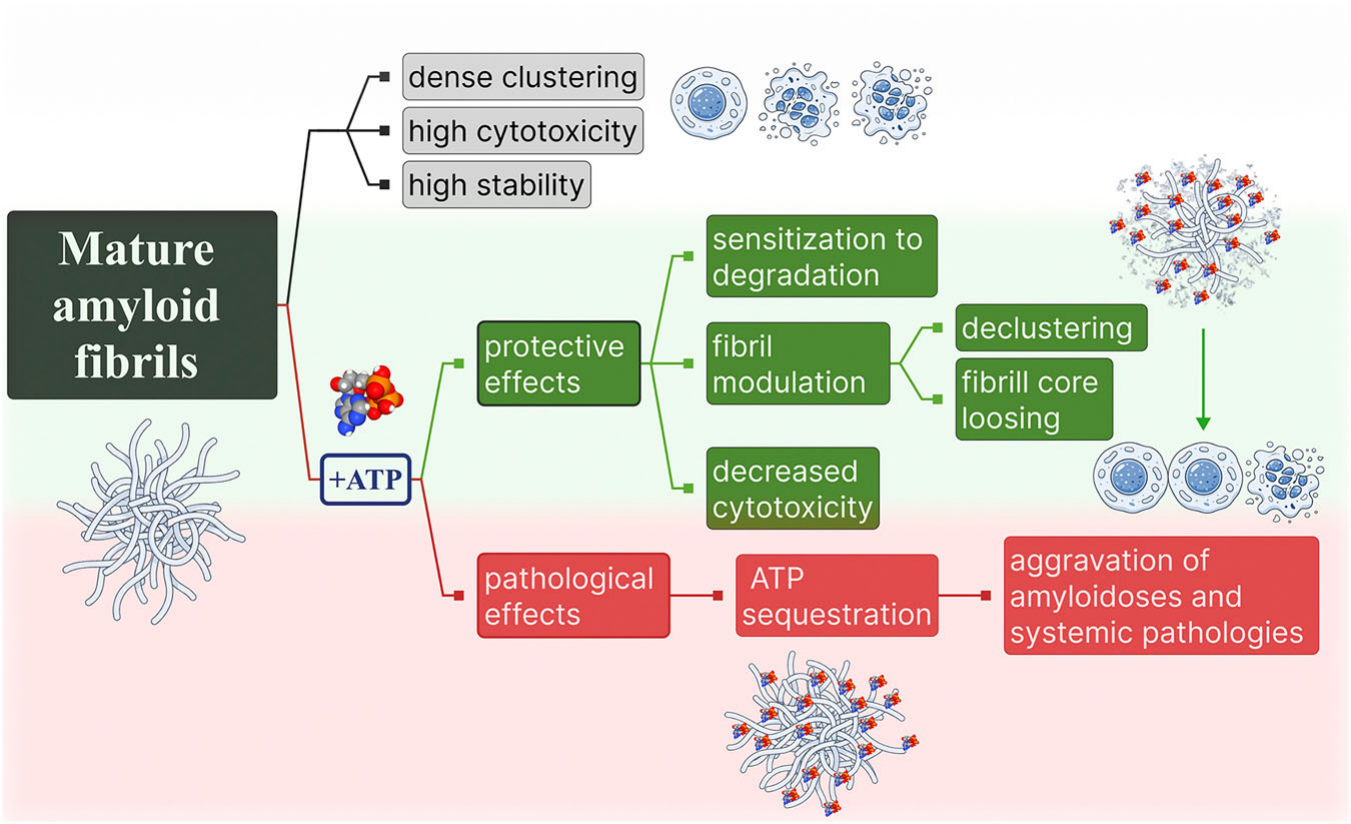
The duality of effects in the interaction between ATP and mature amyloid fibrils. ATP exhibits the low-to-mid micromolar affinity of binding to amyloid fibrils, resulting in both positive (green blocks) and negative (red blocks) consequences: (1) ATP-mediated fibrils remodeling, namely declustering and disordering of aggregates, makes them less cytotoxic and more susceptible to degradation; (2) ATP-sequestration by excessive amyloids exacerbates disorders associated with the depletion of the free nucleotide pool and contributes to amyloidoses and other systemic pathologies progression.

Furthermore, we demonstrated that the declustering and destructuring effects of ATP on lysozyme and sfGFP amyloids are associated with a substantial reduction in their toxic potency to mammalian cells, as well as with a decrease in their resistance to physicochemical degradation processes (Fig. 8).

### The potential consequences of ATP binding to amyloid deposits within the body

The intracellular concentration of ATP is relatively high and ranges from 1 to 10 mМ, depending on the type of tissue and cell [10–13]. It is believed that such excessively high intracellular concentrations of ATP in the millimolar range are due to its participation in the regulation of protein homeostasis in an energy-independent manner [45]. The hydrotropic properties of ATP, due to its amphiphilic nature, determine the ability of this nucleotide to prevent the aggregation of functional proteins, both intrinsically disordered (IDP) and globular, since adenine ring of ATP non-specifically interacts with the hydrophobic regions of proteins and the triphosphate group affects the hydration shell of a protein [15, 46]. In addition, ATP promotes the folding of globular proteins by displacing water molecules that solubilize the unfolded protein and also through its ability to suppress the aggregation of partially folded forms of proteins [47]. At the same time, ATP can establish weak specific interactions with the RNA-binding domains of IDPs, which, along with the dynamic interactions of ATP with positively charged amino acids (arginine/lysine) in the unstructured regions of these proteins, plays an important role in modulating their phase separation [48]. Specific binding of ATP, with an affinity ranging from mid-micromolar to millimolar concentrations, to flexible loop regions of globular proteins and structured domains of IDPs has been shown to regulate the dynamics of these domains, thereby facilitating the stabilization of the native protein form [14, 45, 49].

However, a variety of dysfunctional conditions of the body, encompassing cellular aging [50, 51], neurodegenerative [52], and oncological diseases [53], as well as pre-diabetic states [54], may contribute to a decrease in ATP levels. A number of pathological amyloids are known to form directly in the intracellular environment, including alpha-synuclein aggregates formed in neurons of the brain in Parkinson’s disease [25], intraneural tau tangles in Alzheimer’s disease [27], and cytoplasmic TDP-43 aggregates accumulating in neurons and glial cells in amyotrophic lateral sclerosis, frontotemporal dementia, Alzheimer’s disease, and limbic-predominance age-related TDP-43 encephalopathy [26]. Thus, the ATP concentrations we used, on the order of hundreds of micromoles, do not exceed the limits of possible intracellular availability, especially under conditions of energy stress. Considering the ability of ATP to reduce the cytotoxicity of lysozyme and sfGFP amyloids through their declustering/degradation, as demonstrated in the work, the intracellular functions of this nucleotide apparently go beyond its role as an energy source and its participation in the folding, solubilization, and stabilization of native proteins, but can extend to the protection of cells in amyloidoses (Fig. 8).

As for extracellular ATP, its systemic basal level is strictly controlled by the activity of ectonucleotidases and is in the sub-micromolar range [10, 22, 55]. However, the local microenvironment of different extracellular amyloid deposits represents a heterogeneous biochemical environment in which the micromolar ATP concentrations (up to 250 μM), which we studied, are relevant to both physiological and pathological conditions. First, extracellular amyloid deposits, such as plaques and ganglioside-bound complexes (GAβ), are characterized by preferential localization in the periphery of synapses [56–58]. In this regard, it is important to note that the level of extracellular ATP in the brain is a fluctuating parameter: ATP is released during neurotransmission as a co-transmitter [59, 60], and with intense and repeated stimulation, its local concentration in the synaptic compartment is estimated to exceed 100 µM [61]. Secondly, in pathologies, amyloids can cause cytotoxic damage and disrupt membrane integrity [62–64], which creates conditions for the lysis-dependent release of intracellular ATP into the extracellular space [22, 24]. Concomitantly, Aβ-induced activation of glial cells initiates purinergic signaling involving P2X7 [23, 65, 66], with P2X7 being a low-affinity receptor requiring high micromolar/millimolar concentrations of ATP for its activation [67–69]. The extracellular ATP levels are also elevated in the tumor microenvironment [70]. Taken together, this suggests a plausible scenario for the existence of local extracellular ATP microdomains with high micromolar concentrations near amyloid deposits in pathological processes. Finally, as is known, extracellular amyloid plaques persist in tissues for a long time and contain many “captured” molecules on their surface, which is due to the ability of amyloids to act as an “interactive” matrix capable of accumulating molecules from the microenvironment [62, 71]. Considering the moderate affinity of ATP binding by lysozyme and sfGFP fibrils and extrapolating this property to other amyloids, we can assume that in the late stages of amyloidosis, when pathological aggregates persist in the body for a long time, it is possible for high local concentrations of ATP to accumulate in amyloid plaques (including against the background of the repeated episodes of ATP release described above). This suggests that extracellular amyloid deposits, which are characteristic of systemic amyloidoses, may also be exposed to the declustering and degrading effects of ATP in areas of local inflammation, necrosis, or near a tumor.

Nonetheless, the interplay between ATP and amyloids may not solely result in beneficial outcomes (Fig. 8). The low-to-mid micromolar affinity of lysozyme and sfGFP amyloids fibrils for nucleotides may also be a feature of other amyloid deposits, the accumulation of which could lead to massive sequestration (capture) of free ATP. The situation is further exacerbated by recent findings that have demonstrated that certain intracellular amyloids, such as alpha-synuclein amyloids, are capable of binding and catalytically hydrolyzing ATP [72]. Considering that during neurodegeneration and aging the ATP level is already reduced, its additional binding to amyloids can cause local energy collapse and further destabilize cellular proteostasis [5, 50, 73]. In addition, it has been observed that interactions with lower affinity can reduce the efficiency of the formation of higher affinity complexes [42]. According to the results of this study, when lysozyme and sfGFP undergo a transition to the amyloid state, there is a sharp increase in the ATP binding capacity due to the exposure of additional nucleotide-binding sites. Other amyloid inclusions, accumulating intracellularly or in the extracellular space, can also act as local metabolic “sinks” by binding available nucleotides and thereby affecting the functioning of ATP-dependent systems by disrupting their stronger interaction with the nucleotide (with an affinity in the sub-to-low micromolar range диапазоне [74, 75]), which can contribute to the aggravation of pathology.

In the case of ATP sequestration by extracellular amyloids, this can interfere with the work of purinergic cascades [76], thereby disrupting and exacerbating the function of various human systems and organs. In particular, it is known that purinergic cell communication regulates the functioning of the body’s cardiovascular system and is involved in the work of cellular immunity, development of inflammation and programmed cell death, and its disruption leads to various pathological conditions in the body, including neurodegenerative diseases, heart attacks, strokes, and cancer [67, 77–85].

Since this study represents one of the first to investigate the thermodynamics of ATP binding to mature amyloid fibrils and its cytotoxic consequences, the use of strictly controlled in vitro model systems was imperative. This approach allowed us to successfully separate specific ATP-fibril interactions from tissue-related artifacts (such as non-specific binding of ATP to co-occurring glycosaminoglycans or lipids) and precisely elucidate the underlying biophysical mechanisms of fibril remodeling. However, it is important to acknowledge the inherent limitations of in vitro-assembled aggregates. Recent advances in structural biology, in particular cryo-electron microscopy (cryo-EM), have clearly demonstrated the structural polymorphism of amyloid fibrils grown in vitro and biologically relevant aggregates extracted directly from patient tissues (ex vivo samples) [86, 87]. Since fibril architecture determines the geometry of the ATP binding sites, such polymorphism could potentially modulate the affinity and stoichiometry of ATP interactions. Thus, although our data demonstrate the fundamental possibility of ATP binding to and ATP-mediated remodeling of the model amyloids formed from two globular proteins, namely lysozyme and sfGFP, it is logical and necessary to next validate these mechanisms in disease-associated proteins with an intrinsically disordered structure. Therefore, further studies with a larger sample of different amyloid objects are needed to generalize and confirm the findings obtained using amyloids of lysozyme and sfGFP. Such studies using high-resolution cryo-electron microscopy (cryo-EM) and patient-derived ex vivo samples will be crucial to fully translate these findings into the context of clinical pathology. Furthermore, further analysis of ATP binding to fibrils formed from different proteins and from different sources (e.g., using recently developed ATP-coated gold nanoparticles compatible with atomic force and transmission electron microscopy [88]), as well as obtaining detailed structural information on the nucleotide binding sites in amyloids (e.g., using high-resolution cryo-EM microscopy) may be useful for the development of competitive ATP analogues that could provide its beneficial amyloid declustering effect without the side effects associated with depletion of this valuable nucleotide pool.

In conclusion, it is important to emphasize that in this work we have, for the first time, identified the duality of effects in the interaction between ATP and mature amyloid fibrils of lysozyme and sfGFP. We demonstrate a mid-micromolar affinity of the nucleotide to the fibrillar backbone, which leads to the declustering and disordering of the studied aggregates, accompanied by a reduction in their cytotoxicity. It also results in depletion of the free nucleotide pool with implications for intra- and extracellular processes and potential contribution to amyloidoses and other systemic pathologies progression.

## Materials and methods

### Materials

Thioflavin T (ThT) from AnaSpec (USA), isopropyl-beta-D-1-thiogalactopyranoside (IPTG) from Fluka (Switzerland), buffer components, guanidine hydrochloride (GdnHCl), hen egg white lysozyme (HEWL), adenosine triphosphate (ATP), and 3-(4,5-dimethylthiazol-2-yl)-2,5-diphenyltetrazolium bromide (MTT) from Sigma (USA) were used as received without further purification. Culture flasks were purchased from Jet Biofil (China), flat-bottom 96-well plates were purchased from Kirgen (China).

### Protein production and purification

Superfolder GFP (sfGFP) [89] with poly-histidine tag was expressed and purified as described previously [90]. Briefly, the plasmid pET-28a (+)-sfGFP was transformed into an Escherichia coli BL21(DE3) host (Invitrogen). The sfGFP expression was induced by incubation of the cells with 0.5 mM IPTG during 24 h at 23 °C. The recombinant protein was purified with Ni+-agarose packed in His-GraviTrap columns (GE Healthcare, Sweden). The protein purity was not <95%, as indicated by SDS-PAGE in 15% polyacrylamide gel [91].

### Amyloid fibrils preparation and their interaction with ATP

To obtain lysozyme amyloid fibrils in vitro, the protein at a concentration of 2 mg/ml was incubated for 2 days under intense stirring using the next protocol: in 50 mM KH_2_PO_4_-NaOH buffer in the presence of 3 M GdnHCl, pH 6.3, at 57 °C [92]. Mature amyloid fibrils were dialyzed against Milli-Q water. To obtain sfGFP amyloid fibrils sfGFP in vitro as previously described [35], the protein was incubated at a concentration of 2 mg/ml at 57 °C in 20 mM NaH2PO4/Na2HPO4 (pH 7.4). The constant temperature was maintained by TS-100 Thermo-Shaker (Biosan). Constant agitation was maintained during fibril growth (500 rpm). These highly reproducible protocols allow the production of morphologically identical and previously well-characterized lysozyme and sfGFP amyloids [34, 93] in quantities required for planned studies. The repeatedly obtained fibrils were incubated in the presence of ATP at different concentrations, yielding reproducible results. All samples were included in the analysis.

Before the experiments, amyloid fibrils were collected by centrifugation and resuspended in fresh 20 mM NaH_2_PO_4_/Na_2_HPO_4_ (pH 7.4). A freshly prepared 40 mM ATP-Mg stock solution was prepared in 50 mM NaH_2_PO_4_/Na_2_HPO_4_ (pH 7.4) with pH strictly adjusted to 7.4 and stored at −70 °C. To ensure essential spontaneous ATP hydrolysis does not take place at these conditions, the pH stability of the solutions analyzed was monitored throughout the experiment. To analyze possible non-specific physical effects associated with ATP addition and its possible hydrolysis [38], the influence of NaCl salt on amyloid fibrils was tested.

### Transmission electron microscopy

Amyloid fibrils were visualized using a Libra 120 transmission electron microscope (Carl Zeiss, Germany). Samples were placed onto copper grids coated with formvar/carbon films (Electron Microscopy Sciences, USA) and then stained with a 1% aqueous uranyl acetate solution.

### Confocal microscopy

ThT-stained amyloid aggregates were examined using an Olympus FV 3000 confocal laser scanning microscope (Olympus, Japan). Imaging was conducted with a 60x oil immersion objective lens (numerical aperture 1.42) under 405 nm laser excitation.

### Spectral measurements

A U-3900H spectrophotometer (Hitachi, Japan) was utilized to record the absorption spectra of the samples. To account for light scattering, the absorption spectra of amyloid fibrils and their mixtures with ATP and/or ThT were corrected following a standard protocol [94, 95]. Aggregate size was assessed by measuring the Rayleigh Light Scattering (RLS) at 530 nm.

Fluorescence spectra of the samples were obtained through the Cary Eclipse spectrofluorimeter (Varian, Australia). The spectral slits width did not exceed 5 nm in all experiments. ThT fluorescence was initiated at 440 nm. The concentration of ThT and fibrils from lysozyme and sfGFP was quantified using molar extinction coefficients of *ε*_412_ = 31,600 M⁻¹cm⁻¹ [96], *ε*_280_ = 37,970 M^-1^cm^-1^ [97], and *ε*_280_ = 31,519 M^−1^ cm^−1^ [98], respectively. The absorbance of a dye did not exceed 0.5 in experiments with ThT. Tryptophan fluorescence of analyzed samples was recorded using 295 nm excitation wavelength. The parameter *A* = *I*_320_/*I*_365_, i.e., the ratio of fluorescence intensities on two slopes of the tryptophan fluorescence spectrum, was used to characterize the position and the shape of the tryptophan fluorescence spectra [99]. The anisotropy of tryptophan fluorescence was determined as *r* = (${I}_{V}^{V}\,$− *G*${I}_{H}^{V}$)/ (${I}_{V}^{V}$ + 2 *G*${I}_{H}^{V}$), where ${I}_{V}^{V}$ and ${I}_{H}^{V}$ are vertical and horizontal components of the fluorescence intensity excited by vertically polarized light, respectively, and G = ${I}_{V}^{H}$/${I}_{H}^{H}$ is the coefficient that determines the different instrument sensitivity for the vertical and horizontal components of the fluorescence light, *λ*_em_ = 365 nm [100]. All fluorescence intensity data were corrected for the primary inner filter [101]. Rayleigh light scattering (RLS) was recorded at 530 nm (*λ*ex = 530 nm).

Circular dichroism (CD) spectra were acquired using a J-810 spectropolarimeter (Jasco, Japan). Far-UV CD spectra were collected over the 190–260 nm range with 0.2 nm increments and standard sensitivity, employing a 1 mm path length cuvette. Near-UV CD spectra were collected over the 350–250 nm range with 0.2 nm increments and standard sensitivity, employing a 1 cm path length cuvette. Spectra were scanned at 10 nm/min with an 8 s response time to enhance data quality. Three scans were averaged and baseline-corrected using Milli-Q water as a reference. The secondary structure content of fibrils was estimated using their corrected far-UV CD spectra by the BeStSel web server (https://bestsel.elte.hu) [102, 103]. BeStSel method is believed to predict well the secondary structure of amyloid aggregates and β-structure rich proteins.

### Equilibrium microdialysis and Scatchard curves

Solutions of ATP with amyloid fibrils were prepared through equilibrium microdialysis using a Harvard Apparatus/Amika (USA) system [36, 104]. The procedure was carried out with fibril concentrations of 0.25–1.2 mg/ml and different amounts of ATP. The amount of free ATP was determined by measuring absorbance at 260 nm, applying a molar extinction coefficient of 15,400 M⁻¹cm⁻¹ [105].

Briefly, a sample of protein or amyloid fibrils (*C*_p_) and a nucleotide of known initial concentration (*C*_0_) were placed in two chambers of the device of the same volume (500 μL each). After equilibration, the concentration of free ATP in each chamber will be the same (*C*_f_), while the sample chamber will additionally contain a bound nucleotide (*C*_b_), which can be yielded by equation ${C}_{b}={C}_{0}-2{C}_{f}$. To achieve equilibration of free nucleotide between chambers, samples were incubated for 5 days. The initial concentration of ATP (*C*_0_) and the nucleotide concentration in the sample-free chamber after equilibration (*C*_f_), which were the same in both chambers, were determined by absorption spectrophotometry. In the case of one binding mode the parameters describing the binding of ATP to proteins/amyloid fibrils could be evaluated using the following equation [106]:

$${C}_{b}=\frac{N{C}_{p}{C}_{f}{K}_{b}}{1+{C}_{f}{K}_{b}}$$

where, *K*_b_ is a binding constant and *N* is a number of binding sites. In case of several binding modes the equation is transformed into ${C}_{b}={\sum }_{i}{C}_{{bi}}$. The results were presented as Scatchard curves and fitted in SigmaPlot to determine *K*_b_ and *N* values:

$$\left(\frac{{C}_{b}}{{C}_{p}}\right)/{C}_{f}=N{K}_{b}-{K}_{b}\left(\frac{{C}_{b}}{{C}_{p}}\right)$$

### MTT assay

Human cervical epithelioid carcinoma (HeLa TK-) and neuronal epithelioma SK-N-MC cell lines were sourced from the shared research facility “Vertebrate Cell Culture Collection.” All cell lines were authenticated by STR profiling and were checked for mycoplasma infections. HeLa TK- cells were cultured in complete low glucose Dulbecco’s modified Eagle’s medium (DMEM) medium (Biolot, RF) supplemented with fetal bovine serum (FBS) (10% (v/v) (Global Kang Biotechnology, China). SK-N-MC cells were grown in MEM (Biolot, RF), supplemented with 10% FBS (Global Kang Biotechnology, China). All cell lines underwent authentication via STR profiling and were tested to ensure they were free of mycoplasma contamination. Metabolic activity was assessed 24 hours after treatment with the tested amyloids at a concentration of 0.0015 mg/ml, using the MTT inhibition assay following a previously established standard protocol [95, 107, 108].

### Denatured and pseudo-native sodium dodecyl sulfate (SDS) gel electrophoresis

Denatured SDS–PAGE was carried out following a standard protocol [109, 110]. Prior to loading, samples were mixed with Laemmli SDS–PAGE sample buffer containing 2% SDS (Bio-Rad, Hercules, CA, USA) and boiled for 5 min. For pseudo-native conditions, samples were prepared in a modified buffer (0.0625 M Tris-HCl, pH 6.8, 1% SDS, 10% glycerol, and 0.002% bromophenol blue) and loaded onto the gel without boiling.

### Statistical analysis

All quantitative data are presented as mean ± standard deviation (SD) from five independent measurements (*n* = 5) per group. All the data through experimental groups were validated for their normal distribution by the Shapiro–Wilk test and the homogeneity of the variances in each group by Bartlett’s test. To compare experimental groups (amyloid aggregates in the presence of ATP) and the control group (intact amyloids), ANOVA with post hoc test according to the Dunnett’s procedure was applied. A *p*-value < 0.01 was considered statistically significant, corresponding to the 99% confidence level. For transparency, *p*-values < 0.05 are also reported and annotated in the figures as indicators of moderate trends. Significance levels are indicated in the results as follows: *p* < 0.001 (***), *p* < 0.01 (**), *p* < 0.05 (*), and *p* ≥ 0.05 (ns). Statistical analyses were performed using GraphPad Prism version 10 (GraphPad Software, San Diego, California, USA).

## Supplementary information

Supplementary file.docx

## Data Availability

All analyzed and generated data during current study are included in the article and Supplementary information. Further information is available from the corresponding author on reasonable request.
