# Supplementary material for: ATP binding to lysozyme and superfolder GFP amyloid fibrils induces aggregate remodeling and attenuates their cytotoxicity
Source: Cell Death Discov. 2026 Jun 4;12:330. doi: 10.1038/s41420-026-03186-9 (PMC13451496; doi:10.1038/s41420-026-03186-9)
Supplement: Supplementary file 1 [file 41420_2026_3186_MOESM1_ESM.docx]

Supplementary Information

**ATP binding to lysozyme and superfolder GFP amyloid fibrils induces aggregate remodeling and attenuates their cytotoxicity**

**Olga V. Stepanenko^1,^** ^†^**, Maksim I. Sulatsky^2,^** ^†^**, Kristina G. Gridasova^1^, Ekaterina V. Mikhailova^1^, Anna I. Sulatskaya^1^, Olesya V. Stepanenko^1,*^**

^1^Laboratory of structural dynamics, stability and folding of proteins, Institute of Cytology Russian Academy of Sciences, 4 Tikhoretsky ave., 194064 St. Petersburg, Russia; sov@incras.ru; krgridasova@yandex.ru; 4evamkh@gmail.com; ansul@mail.ru; lvs@incras.ru

^2^ Laboratory of cell morphology, Institute of Cytology Russian Academy of Sciences, 4 Tikhoretsky ave., 194064 St. Petersburg, Russia; [m_sulatsky@mail.ru](mailto:m_sulatsky@mail.ru)

^†^These authors contributed equally to the work

*****Corresponding author: Olesya V. Stepanenko, Laboratory of structural dynamics, stability and folding of proteins, Institute of Cytology Russian Academy of Sciences, 4 Tikhoretsky ave., 194064 St. Petersburg, lvs@incras.ru

**Figure S1. Invariance of morphology of amyloid fibrils during the experiment.** (***a***) Transmission electron microscopy (TEM) images of control amyloid samples (in the absence of ATP) at the beginning (start) and end (end) of the experiment studying the interaction of ATP with AF-lys7 and AF-sfGFP amyloids. Scale bars are equal to 1 µm. (***b***) The effect of the order of ATP and ThT addition to lysozyme amyloids on ThT fluorescence intensity: first ATP, then ThT (AF-lys7, +ThT, +ATP), first ThT, then ATP (AF-lys7, +ATP, +ThT). λ_ex_ = 440 nm.

**Figure S2. Invariance of morphology and structure of amyloid fibrils with changes in ionic strength.** The data for AF-lys7 (***a***–***f***) and AF-sfGFP (***g***–***l***) in the absence and in the presence of 1 mM of NaCl are presented. (***a*** and ***g***) Transmission electron microscopy (TEM) of AF-lys7 and AF-sfGFP. Scale bars are equal to 1 µm. (***b*** and ***h***) Confocal laser scanning microscopy (CLSM) of AF-lys7 and AF-sfGFP: an overlay of fluorescence of ThT added to amyloid samples and transmitted light images are presented. Scale bars are equal to 20 µm. (***c*** and ***i***) Far-UV CD spectra of AF-lys7 and AF-sfGFP. (***d***) Parameter *A* (ratio of fluorescence intensity at 320 and 365 nm wavelengths, the value of which is sensitive to the microenvironment of tryptophan residues) and fluorescence anisotropy (λ_em_ = 365 nm) of AF-lys7, λ_ex_ = 295 nm. (***e*** and ***k***) Integral fluorescence intensity of ThT added to samples of AF-lys7 and AF-sfGFP, λ_ex_ = 440 nm. (***j***) Fluorescence spectra of aromatic residues and the specific chromophore of AF-sfGFP, λ_ex_ = 280 nm. (***f*** and ***l***) The value of Rayleigh Light Scattering (RLS) of AF-lys7 and AF-sfGFP. Data in bar graphs are presented as mean ± SD, based on five replicates

**Figure S3. Fluorescence of ThT dye under different conditions.** Integral fluorescence intensity of free ThT in the solution with or without ATP at a concentration of 250 μM. For the comparison, the values of integral fluorescence intensity of fibril-bound ThT in the AF-lys7 and AF-sfGFP samples, untreated or exposed to 250 μM ATP, are shown. λ_ex_ = 440 nm.

**Figure S4. Cytotoxicity of** **ATP solutions**. Relative metabolic activities of cervical epithelioid carcinoma (HeLa TK-) cell line are presented after 24 h exposure to ATP solution at a concentration of up to 500 μM. All values are normalized to the metabolic activity of control cells treated with non-fibrillar protein. Data are presented as mean ± SD, based on five replicates.
